# Metabolite Profiling of Dihydroartemisinin in Blood of *Plasmodium*-Infected and Healthy Mice Using UPLC-Q-TOF-MS^E^

**DOI:** 10.3389/fphar.2020.614159

**Published:** 2021-01-18

**Authors:** Yifan Zhao, Peng Sun, Yue Ma, Xiaoqiang Chang, Xingyu Chen, Xin Ji, Yue Bai, Dong Zhang, Lan Yang

**Affiliations:** ^1^ Institute of Chinese Materia Medica, China Academy of Chinese Medical Sciences, Beijing, China; ^2^ Artemisinin Research Center, China Academy of Chinese Medical Sciences, Beijing, China

**Keywords:** dihydroartemisinin (DHA), metabolite profiling, UPLC-ESI-Q-TOF-MS^E^, UNIFI platform, *Plasmodium berghei* ANKA, blood, plasma, red blood cell

## Abstract

Dihydroartemisinin (DHA) and its’ derivatives have been employed as the most powerful first-line drugs for malarial treatment for several decades. The metabolism of DHA has not been studied clearly. Previous reports were focused on the pharmacokinetics procedure of DHA in healthy rats. The metabolites of DHA in red blood cells (RBC), especially in the RBC from *Plasmodium*-infected models, have rarely been studied. The *Plasmodium* species parasitize inside RBC, and these cells should be the final place where DHA performs its activity. In this study, the profile of DHA metabolites in biosample (blood, plasma, and RBC) of the infected and healthy mice was investigated with UPLC-Q-TOF-MS and UNIFI platform to gain insight into DHA metabolism. Results show that a total of 25 metabolites were successfully identified in infected (30 in healthy) blood, 27 in infected (27 in healthy) plasma, and 15 in infected (22 in healthy) RBC. Results show that hydroxylation, OH-dehydration, and glucuronidation reactions were important in the metabolic pathway *in vivo*. Significantly, DHA metabolites inside RBC were identified for the first time. 8-Hydroxy (8-OH) DHA, 4*α*-OH deoxy ART, and 6*β*-OH deoxy ART were identified *in vivo* for the first time.

## Introduction

**FIGURE 1:**
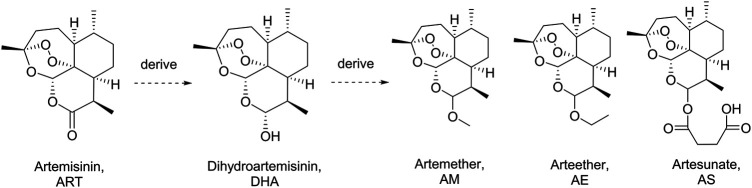
Artemisinin, dihydroartemisinin, and the derivatives.

Artemisinin (ART), a sesquiterpene lactone containing a peroxide group, was first discovered by Tu et al. in 1972. It is derived from the leaves of *Artemisia annua* L. (qinghao) and displays highly antimalarial activity. Later, a more powerful dihydroartemisinin (DHA) was developed by Tu in 1973 by introducing a hydroxyl group into the structure of ART (Tu et al., 1981). Nowadays, a series of DHA derivatives, such as DHA, artemether, arteether, and artesunate, were developed to lay the foundation of the first-line antimalarial treatment all over the world (Figure 1) (Hou and Huang, 2016; Gutman et al., 2017). The pharmacokinetics procedure of DHAs is one of the most concerning issues for pharmaceutical scientists due to their great clinical applications (Karunajeewa, 2011). Previous studies proved that the drugs derived from DHA could be transformed into DHA *in vivo* rapidly (Lee and Hufford, 1990; Chi et al., 1991; De Vries and Dien, 1996). Moreover, DHAs can be cleared in a short half-life of 2.4 h, which was contradictory to their long duration of action (Tu, 2009). Thus, the metabolites of DHA could be of great importance in its pharmacological activity.

Early studies have shown that most DHAs were metabolized into metabolites *in vivo* by radioactive thin-layer chromatography (Tu, 2009). Representatively, Maggs (Maggs et al., 1997), Ilett (Ilett et al., 2002), and Liu (Liu, 2012) identified dozens of DHA metabolites in plasma, bile, urine, and feces from healthy rats through liquid chromatography-mass spectrometry (LC-MS). However, *Plasmodium*-infected models have been rarely studied. Moreover, the *Plasmodium* species parasitize inside red blood cells (RBC), and these cells are the final place where DHA performs its activity. Early studies have shown that radioisotope-labeled 3H-DHA accumulates from the culture medium into healthy and infected RBC (h-RBC and i-RBC); especially, the partitioning of radioactivity inside i-RBC was >300-fold higher than the concentration measured in the medium (Gu et al., 1984). However, the detection of DHA metabolites in RBC is a great challenge because the inner environment of RBC is very complex and the peroxide group of DHA and metabolites are markedly sensitive to iron ions or heme, particularly in organic solvents (Sibmooh et al., 2001; Moles et al., 2008). Up to now, the structures of the active metabolite of DHA remain unclear.

Our group has been devoted to studying ART drugs for decades (Tu, 2011; Bai et al., 2019; Ma et al., 2019; Zhang et al., 2019). In this study, a C57 mouse model infected by *Plasmodium berghei* ANKA strain was established to reveal the real metabolic process of DHA. The profiles of DHA metabolites in biosample (blood, plasma, and RBC) of infected and healthy mice were investigated with UPLC-Q-TOF-MS to obtain insight into DHA metabolism. A combination of MS^E^ data acquisition technology and the integrated data processing functions of the UNIFI platform was applied for the rapid detection and structural characterization of unknown metabolites. Furthermore, DHA metabolites inside RBC were firstly identified from blood and RBC of mouse. This study provides the experimental basis for further exploration of the antimalarial mechanism of DHA, particularly inside RBC.

## Materials and Methods

### Materials and Reagents

ART and DHA were obtained from the Chongqing Wuling Mountain Pharmaceutical, Kunming Pharmaceutical Group (Chongqing, China, the batch numbers are MFCD00081057 and C00220160402, resp.). The compound had a purity of ≥99%. 3*α*-Hydroxy deoxy DHA was provided by Professor J.Y. Wang (China Academy of Chinese Medical Sciences, Beijing, China). The reagents used were of high-performance liquid chromatography (HPLC) grade. Acetonitrile and methanol were purchased from Fisher (Fisher Chemical, Geel, Belgium); formic acid and ethyl acetate were purchased from Beijing Chemical Works (Beijing, China). Sodium pentobarbital was purchased from Merck (Darmstadt, Germany). Water was prepared using a Milli-Q system operating at 18.2 MΩ (Millipore, Bedford, MA, United States). All other chemicals used were purchased from Fisher Scientiﬁc or Beijing Chemical Works and were of the highest purity available. Potassium dichromate was obtained from Sinopharm Chemical Reagent Co., Ltd (Beijing, China). Ethylenediaminetetraacetic acid disodium salt (EDTA-Na_2_) was purchased from Beijing Solaria Science & Technology Co., Ltd (Beijing, China).

### Instruments and LC-MS Conditions

The UPLC-ESI-Q-TOF-MS^E^ system consisted of a Waters ACQUITY I-class UPLC and Xevo G2-XS Q-TOF Mass Spectrometer (Waters Corporation, Milford, MA, United States), equipped with an electrospray ionization (ESI) source (Waters Corporation, Milford, MA, United States). Chromatographic separation was achieved using an ACQUITY UPLC BEH C_18_ (2.1 mm × 100 mm, 1.7 µm, Waters). The mobile phase consisted of solvent A (H_2_O containing 0.1% formic acid, *v/v*) and solvent B (acetonitrile containing 0.1% formic acid, *v/v*). The gradient program for biosamples included three segments: 5%–100% B from 0 to 15 min, followed by 100%–5% B from 15 to 17 min, followed by a postrun of 3 min for column equilibration. The ﬂow rate was 0.4 ml/min, and the temperature was at 30°C throughout the analysis.

The MS was operated in positive ionization mode across a scan range of *m/z* 50 to 1,000, with a scan time of 0.2 s. Source parameters were as follows: source temperature, 150°C; cone gas, 50 L/h; desolvation temperature, 450°C; desolvation gas ﬂow, 800 L/h. Argon (99.95%) was used for collision-induced dissociation, and N2 was used as the drift gas. The low collision energy was set to 6 eV, and the high collision energy was ramped from 12 to 25 eV. MS^E^ analysis was employed for simultaneous acquisition of the exact mass of small molecules at high and low collision energy. The MS was calibrated daily by Waters calibration standard. Leucine enkephalin was used for lock mass correction with *m/z* 556.2771 at an interval of 0.5 min to ensure robust and accurate mass measurements.

### MS Data Processing

In this analysis, data acquisition was performed with the MS^E^ scan function using MassLynx4.1 (Waters Corporation, Milford, MA, United States). The components were identiﬁed in a nontargeted manner by spectral deconvolution in UNIFI 1.9 (Waters Corporation, Milford, MA, United States) with the following parameters: three-dimensional peak detection mode; low-energy limits of 150 counts and high-energy limits of 20 counts; isotope clustering; drift peak with a mass accuracy of ±2 mDa; the maximum number of allowed fragment ions per match set at 10. Furthermore, the detection of metabolites was accomplished using data processing tools, such as mass defect filter, common fragment filter, common neutral loss fragment filter, and binary comparison.

### Animals

Male C57BL/6J mice (weighing 18–22 g) were supplied by Beijing Vital River Laboratory Animal Technology Corporation (Beijing, China; Grade SPF, Certificate No. SCXK 2019–0003). The experimental protocol was approved by the Ethics Committee of the China Academy of Chinese Medical Sciences (ethics approval number: 2019080). Mice were randomly divided into a healthy group (H, n = 5) and an infected group (I, n = 5). Corresponding control groups were set up separately (n = 2). Mice were fasted for 12 h prior to drug administration and for an additional 1 h after dosing. The mice had ad libitum access to water during the experiments.

### Parasite Recovery and Infection Procedures

The rodent malaria parasite *Plasmodium berghei* ANKA strain (chloroquine-sensitive) was stored at Artemisinin Research Center, China Academy of Chinese Medical Sciences (Beijing, China). A vial of *Plasmodium berghei* ANKA was retrieved from liquid nitrogen storage, thawed in a water bath at 37°C, and intraperitoneally inoculated into two mice. Subsequent passage to maintain the parasites was performed by intraperitoneal inoculation of a normal mouse with infected blood (i-blood) from a donor mouse. I-blood was diluted with sterile, nonpyrogenic 0.85% saline to provide 1 × 10^7^ i-RBC in an injection volume of 0.2 ml. The experiment was only performed when the levels of parasitemia reached 30%.

#### Measurement of Parasitemia

A drop of i-blood was collected from the mouse by venesection of the tail and transferred onto the edge of a microscope slide (single, 76 × 26 mm thickness). The blood was drawn evenly across a second slide to produce a thin blood film, fixed with methanol, and then allowed to dry at room temperature before staining with Giemsa’s stain solution (Sigma, Saint Louis, Missouri, United States). The slides were viewed using light microscopy (Olympus, Tokyo, Japan) with oil immersion (1,000× magnification). The percentage of parasitemia was calculated by counting the number of i-RBC in 1,000 cells in a thin blood film.

### Animal Handling and Sample Preparation

DHA was formulated as a suspension in 0.5% sodium carboxymethylcellulose. Groups H and I (n = 5) received a single oral dose of DHA (200 mg/kg). The blank control received 0.5% sodium carboxymethylcellulose only. The blood samples were collected into EDTA tubes at 1.0 h after administration by eye removal under anesthesia state (sodium pentobarbital; 45 mg/kg *bw*; intraperitoneally). Subsequently, all mice were sacrificed by cervical dislocation. This time point was selected based on a preliminary study showing that t_max_ of DHA in blood was 0.5 h and further drug metabolism also requires some time. Plasma and RBC were separated by centrifugation (Fresco 21, Thermo Fisher, Osterode, Germany) at 3,500 rpm for 15 min, at 4°C. In this study, potassium dichromate was employed to deactivate the Fe^2+^ ion in hemoglobin in blood samples, while ethylenediaminetetraacetic acid disodium salt (EDTA-Na_2_) could simultaneously chelate Fe^2+^ and Fe^3+^ to protect the peroxide group of DHA (Lindegardh et al., 2011; Dai et al., 2019). An aliquot of 150 μL of blood, plasma, or RBC was added to a 1.5 ml centrifuge tube and immediately spiked with 30 μL of potassium dichromate 0.4 M and 50 μL of EDTA-Na_2_ (3%, m/v). The mixture was vortex-mixed for 1 min on ice. After freezing at −80°C and rapid thawing at 37°C for three freeze-thaw cycles, the mixture was subjected to ultrasonication for 5 min in ice water. The supernatants of samples were loaded onto pretreated SPE cartridges (Oasis PRiME HLB 1 cc Extraction Cartridges; Waters Corporation). The cartridges were washed with 1 ml of water, and analytes were eluted with 1 ml of methanol. The methanol fractions were dried using a vacuum freeze concentrator (Ji‘aimu Corporation, Beijing, China). All samples were stored at −80°C until reconstitution with 100 µL of the initial mobile phase and immediate analysis. Aliquots (4 µL) of the reconstituted solutions were injected into the LC-MS.

## Results

### Optimization of Sample Preparation

Protein precipitation, liquid-liquid extraction, and solid-phase extraction (SPE) were evaluated by the extraction of metabolites. Protein precipitation was achieved by adding four times the volume of acetonitrile to remove the protein (Qing et al., 2014). Liquid-liquid extraction was achieved by adding 1.0 ml of ethyl acetate to extract the target compounds (Dai et al., 2019). The elution of SPE was achieved by loading the sample on an SPE column, followed by the addition of 1.0 ml of water, and subsequently 1.0 ml of methanol at the flow rate of one drop per 3 s (Ma et al., 2019). In addition, the target metabolites were gathered in the organic phase. As a result, SPE allowed for the simultaneous extraction of more target metabolites, particularly phase II metabolites.

### Identification of Reference Substances

The reference substances DHA, ART, and 3*α*-hydroxy (3*α*-OH) deoxy DHA were used to establish the standard MS fragmentation patterns.

**FIGURE 2:**
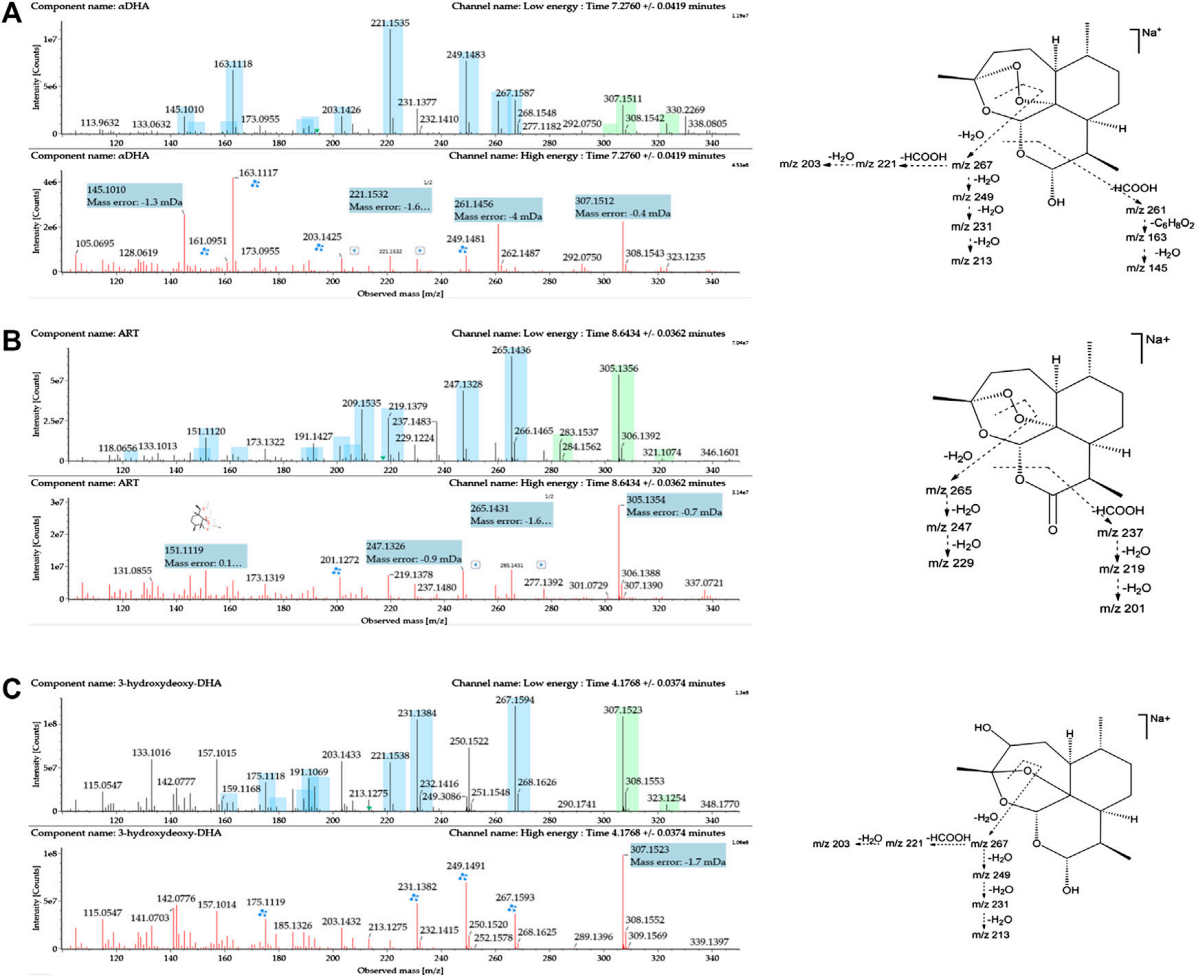
Mass spectrometry (MS^E^) spectra of **(A)** DHA, **(B)** ART, and **(C)** 3*α*-hydroxy deoxy DHA and the proposed MS fragmentation pathways. A blue box behind the low-energy fragment ions shows in-source fragments, whereas a green box indicates adduct clusters.

DHA was eluted at 7.28 min. The quasimolecular ions were *m/z* 307.1515 [M + Na]^+^ and *m/z* 323.1251 [M + K]^+^. Fragments *m/z* 267.1597, 249.1491, 231.1387, and 213.1282 were generated by the successive loss of H_2_O from *m/z* 307.1515. Fragment *m/z* 221.1543 was generated by the loss of HCOOH from *m/z* 267.1597. Moreover, fragment *m/z* 261.1476 was generated by the loss of HCOOH from *m/z* 307.1515. Fragments *m/z* 163.1125 and 145.1021 were generated by the successive loss of C_6_H_8_O_2_ and H_2_O from *m/z* 261.1476. Mass spectra and the MS fragmentation pattern of DHA are shown in Figure 2A.

ART was eluted at 8.64 min. The quasimolecular ions were *m/z* 283.1545 [M + H]^+^, *m/z* 305.1361 [M + Na]^+^, and *m/z* 321.1092 [M + K]^+^. Fragments *m/z* 265.1447, 247.1335, and 229.1230 were obtained by successive loss of H_2_O from *m/z* 283.1545. Moreover, the fragment ion at *m/z* 237.1490 was generated by the loss of HCOOH from *m/z* 283.1545. Fragments *m/z* 219.1386 and 201.1281 were generated by the successive loss of H_2_O from *m/z* 237.1490. Mass spectra and the MS fragmentation pattern for ART are shown in Figure 2B.

3*α*-OH deoxy DHA was eluted at 4.17 min. The quasimolecular ions were *m/z* 307.1540 [M + Na]^+^ and 323.1265 [M + K]^+^. The MS fragmentation pattern was similar to that of DHA. Fragments *m/z* 267.1602, 249.1534, 231.1395, and 213.1285 were generated by the successive loss of H_2_O from *m/z* 307.1540. Fragments *m/z* 221.1546 and 203.1441 were generated by the successive loss of HCOOH and H_2_O from *m/z* 267.1602. Mass spectra and the MS fragmentation pattern for 3*α*-OH deoxy DHA are shown in Figure 2C.

### Identification of Metabolites

To evaluate the metabolism of DHA *in vivo*, metabolites in blood, plasma, and RBC of infected and healthy mice were acquired by UPLC-electrospray ionization-Q-TOF MS operating in MS^E^ mode and identified using the UNIFI 1.9 platform (Waters Corporation, Milford, MA, United States). Metabolites were identified based on retention time and characteristic behavior of MS, including the exact mass, quasimolecular ions, in-source fragmentation, and characteristic fragments of metabolites. Compared with the peaks in the corresponding blank sample, a total of 34 peaks were observed and identified as DHA metabolites in blood samples of infected and healthy mice.

**FIGURE 3:**
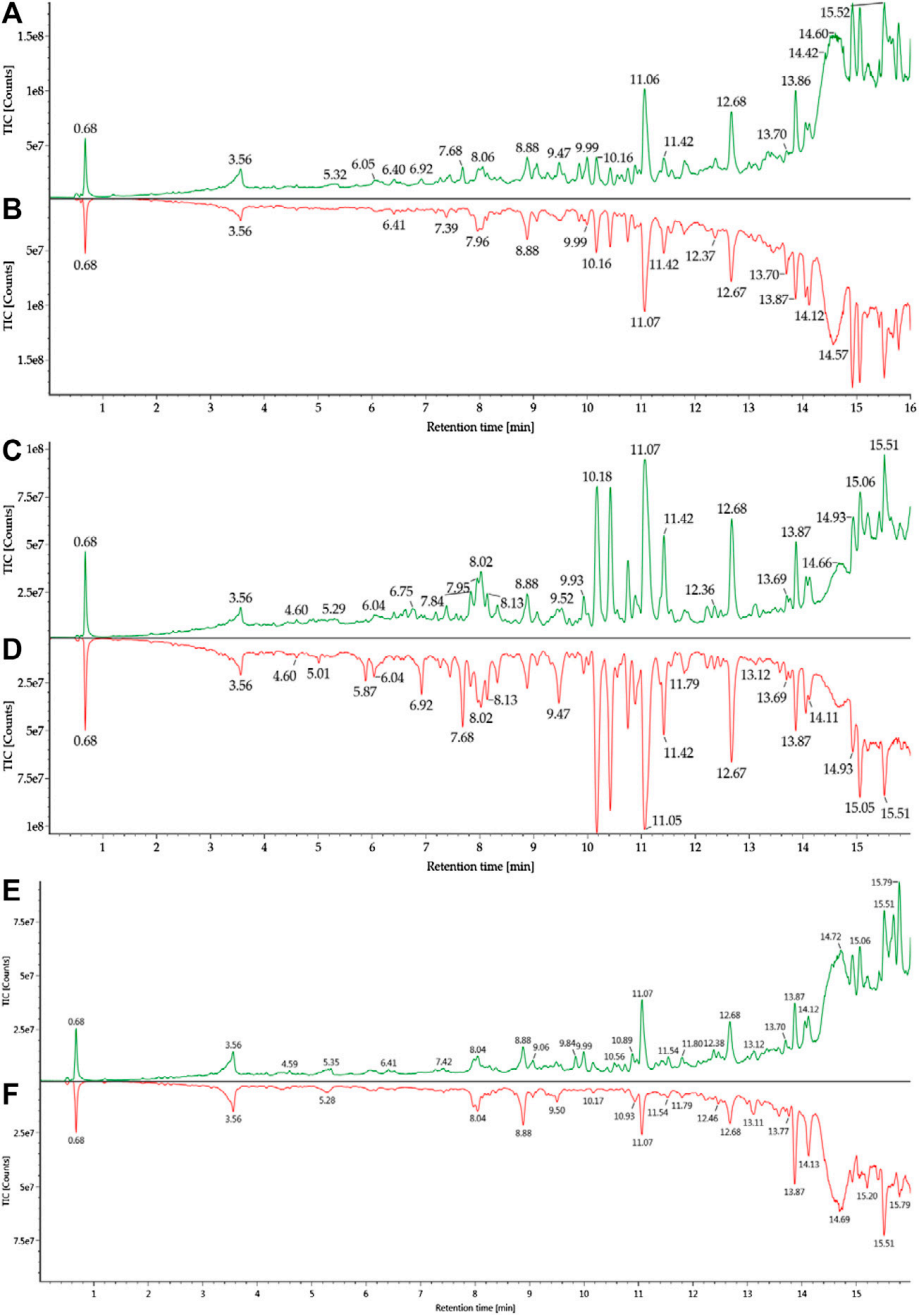
Total ion chromatograms of blood samples from healthy and infected mice: **(A)** h-blood; **(B)** i-blood; **(C)** h-plasma; **(D)** i-plasma; **(E)** h-RBC; **(F)** i-RBC.

**FIGURE 4:**
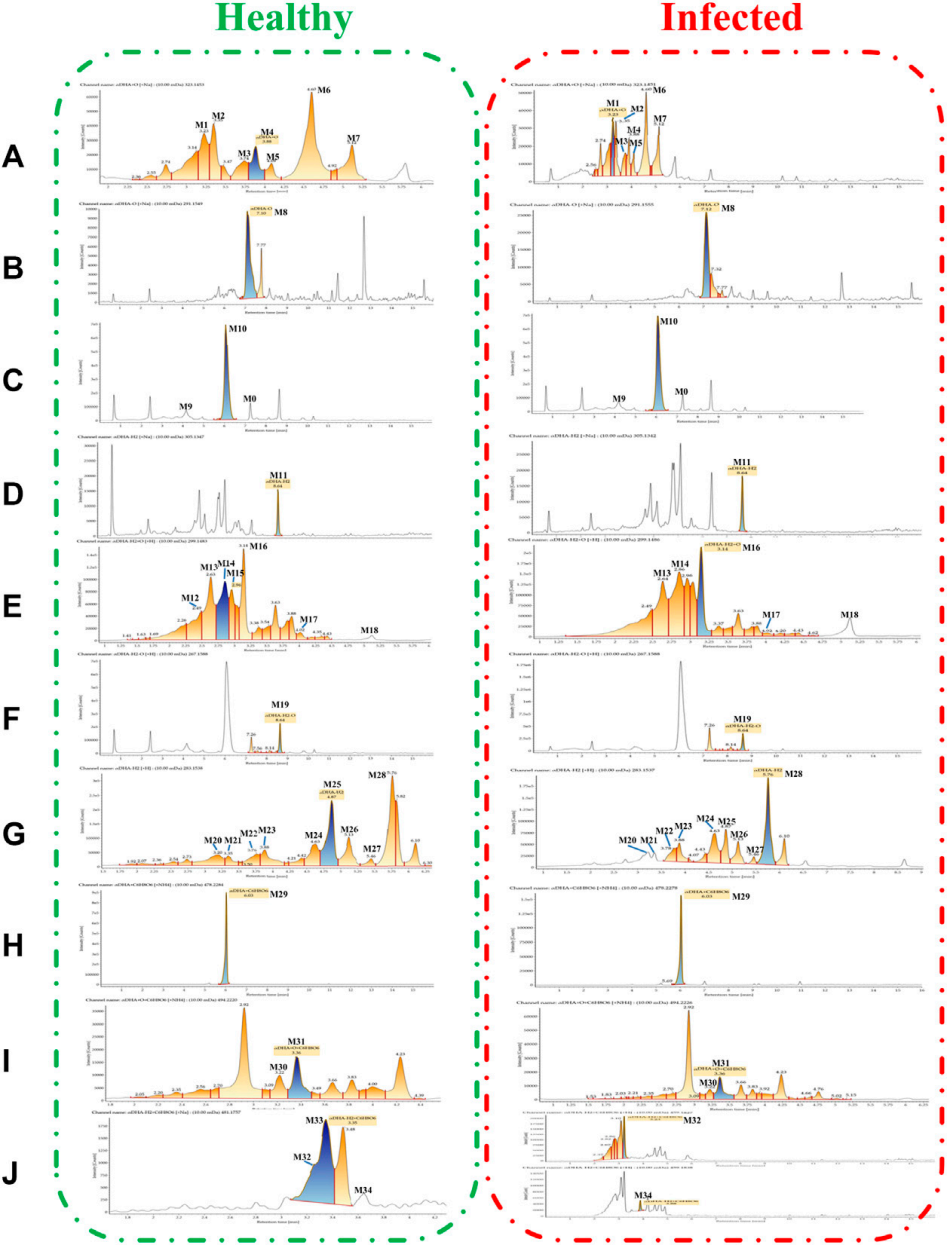
Extracted ion chromatograms of DHA metabolites obtained from healthy blood samples (left) and infected blood samples (right): **(A)** DHA + O (M1–M7); **(B)** DHA-O (M8); **(C)** DHA-O + O (M9, M10); **(D)** DHA-H_2_ (M11); **(E)** DHA-H_2_+O (M12–M18); **(F)** DHA-H_2_-O (M19); **(G)** DHA-H_2_-O + O (M20–M28); **(H)** DHA + C_6_H_8_O_6_ (M29); **(I)** DHA + O + C_6_H_8_O_6_ (M30, M31); **(J)** DHA-H_2_+C_6_H_8_O_6_ (M32–M34).

The metabolites were generated by multiple enzymatic reactions, such as monohydroxylation, deoxygenation, OH-deoxygenation, dehydrogenation, OH-dehydrogenation, dehydration, OH-dehydration, glucuronidation, dehydro-glucuronidation, and OH-glucuronidation. All identified DHA metabolites are listed in Table 1. Total ion chromatograms of blood samples from infected and healthy mice are shown in Figure 3. Extracted ion chromatograms of the metabolites in blood, plasma, and RBC are shown in Figure 4.

**TABLE 1:** Summary of metabolites of DHA detected in the blood, plasma, and RBC of infected and healthy mice.

Metabolites	Metabolic pathways	Observed m/z	RT (min)	Mass error (mDa)	Adducts	i-blood	i-plasma	i-RBC	h-blood	h-plasma	h-RBC	Major fragments	Identification by standards
M0	P	307.1511	7.28	−0.5	+Na, +NH4, +K	+	+	+	+	+	+	323.12; 307.15; 302.20; 267.16; 261.15; 249.15; 231.14; 221.15; 203.14; 163.11	DHA
M1	P + O	323.1450	3.22	−1.5	+Na, +K	+	+	+	+	+	+	339.12; 323.15; 283.15; 265.14; 247.13; 219.14; 201.13; 179.11; 161.10	
M2	P + O	323.1448	3.35	−1.7	+Na, +K	+	+	+	+	+	+	339.12; 323.15; 283.15; 265.14; 247.13; 219.14; 179.11; 161.10	
M3	P + O	323.1455	3.71	−1.0	+Na, +K	+	+	−	+	+	+	323.14; 283.15; 265.14; 247.13; 219.14; 201.13; 179.11; 161.10	
M4	P + O	323.1444	3.87	−2.1	+Na, +K	+	+	−	+	+	+	339.12; 323.15; 283.15; 265.14; 247.13; 219.14; 201.13; 179.11; 161.10	8 OH DHA
M5	P + O	323.1438	4.08	−2.7	+Na, +K	+	+	−	+	−	+	339.12; 323.15; 283.15; 265.14; 247.13; 219.14; 201.13; 179.11; 161.10	
M6	P + O	323.1450	4.59	−1.5	+Na, +NH4, +K	+	+	+	+	+	+	339.12; 323.15; 318.19; 283.15; 265.14; 247.13; 237.15; 219.14; 179.11; 161.10	
M7	P + O	323.1444	5.11	−2.1	+Na, +K	+	+	+	+	+	+	339.12; 323.15; 283.15; 265.14; 247.13; 219.14; 201.13; 179.11; 161.10	
M8	P-O	291.1546	7.09	−2.1	+Na	+	+	−	+	+	+	307.13; 291.16; 251.16; 233.15; 205.16	1 deoxy DHA
M9	P-O + O	307.1500	4.17	−0.4	+Na	+	+	+	+	+	+	323.12; 307.15; 267.16; 249.15; 231.14; 221.15; 203.14	3α-OH deoxy DHA
M10	P-O + O	323.1206	6.04	−4.9	+K	+	+	+	+	+	+	323.15; 307.15; 267.16; 239.16; 231.14; 203.14	
M11	P-H2	305.1336	8.63	−2.3	+Na, +NH4	+	−	−	+	−	−	305.13; 283.15; 265.14; 247.13; 219.14; 201.13	ART
M12	P-H2+O	299.1482	2.48	−0.7	+H, +Na	−	−	−	−	+	−	321.13; 299.15; 281.14; 263.13	
M13	P-H2+O	299.1476	2.63	−1.2	+H, +Na, +K	−	+	−	−	+	−	337.10; 321.13; 299.15; 281.14; 263.13; 215.12	
M14	P-H2+O	299.1476	2.85	−1.3	+H, +NH4, +Na	+	+	+	+	+	+	337.10; 321.13; 316.17; 299.15; 281.14; 263.13	
M15	P-H2+O	299.1481	2.96	−0.8	+H	−	−	−	+	−	−	321.13; 299.15; 281.14; 263.13; 205.12; 155.07	
M16	P-H2+O	299.1477	3.14	−0.6	+H, +Na	+	+	+	+	+	+	321.13; 299.15; 281.14; 263.13; 217.12	
M17	P-H2+O	299.1471	4.03	−1.8	+H	+	+	−	+	+	−	337.10; 321.13; 316.18; 281.14; 263.13; 253.14; 235.13; 223.13; 163.11; 149.10	
M18	P-H2+O	321.1298	5.10	−1.0	+Na	+	−	−	+	−	−	321.13; 281.14; 263.13; 235.13; 217.12	
M19	P-H2-O	267.1578	8.64	−1.3	+H, +Na	+	+	+	+	+	+	305.11; 289.14; 267.16; 249.15; 239.16; 231.14; 221.15; 207.14; 203.14	1 deoxy ART
M20	P-H2-O + O	283.1511	3.21	−2.8	+H	+	+	−	+	+	+	283.15; 265.14; 247.13; 219.14; 201.13; 179.11; 161.10	
M21	P-H2-O + O	283.1491	3.35	−0.9	+H	−	−	+	+	−	+	283.15; 265.14; 247.13; 237.15; 219.14; 201.13; 179.11; 161.10	
M22	P-H2-O + O	283.1526	3.74	−1.4	+H	+	+	−	+	+	+	305.13; 283.15; 265.14; 247.13; 237.15; 219.14; 179.11; 161.10	
M23	P-H2-O + O	283.1533	3.87	−0.7	+H	+	+	−	+	+	+	283.15; 265.14; 247.13; 219.14; 201.13; 179.11; 161.10	
M24	P-H2-O + O	283.1533	4.61	−0.6	+H, +Na	+	+	−	+	+	−	305.13; 283.15; 265.14; 247.13; 237.15; 219.14; 201.13; 179.11; 161.10	
M25	P-H2-O + O	283.1534	4.87	−0.5	+H, +Na	+	+	+	+	+	+	321.11; 305.13; 283.15; 265.14; 247.13; 237.15; 219.14; 201.13	
M26	P-H2-O + O	283.1524	5.12	−1.6	+H, +Na	+	+	+	+	+	+	305.13; 283.15; 265.14; 247.13; 219.14; 201.13; 179.11; 161.10	4α-OH deoxy ART
M27	P-H2-O + O	283.1528	5.45	−1.1	+H, +NH4, +Na	−	+	−	−	+	−	305.13; 300.17; 283.15; 265.14; 247.13; 229.14; 219.14; 201.13	
M28	P-H2-O + O	283.1531	5.75	−0.9	+H	+	+	+	+	+	+	305.13; 300.18; 283.15; 265.14; 247.13; 229.14; 219.14; 201.13	6β-OH deoxy ART
M29	P + C6H8O6	478.2235	6.03	−4.8	+NH4	+	+	+	+	+	+	478.23; 267.16; 249.15; 231.14; 221.15; 203.14; 191.11; 163.11	
M30	P + O + C6H8O6	494.2211	3.21	−2.1	+NH4	−	+	−	+	+	−	499.18; 494.22; 339.12; 323.15; 283.15; 265.14; 247.13; 219.14; 201.13	
M31	P + O + C6H8O6	494.2226	3.37	−0.6	+NH4, +Na	−	+	−	+	+	−	499.18; 494.22; 339.12; 323.15; 283.15; 265.14; 247.13; 237.15; 219.14	
M32	P-H2+C6H8O6	459.1831	3.23	−3.0	+H	+	−	+	+	−	+	459.18; 283.15; 265.14; 247.13; 237.15; 219.14	
M33	P-H2+C6H8O6	481.1735	3.35	5.5	+Na	−	−	−	+	−	−	481.17; 283.15; 265.14; 247.13; 237.15; 219.14	
M34	P-H2+C6H8O6	459.1838	3.88	−2.2	+H	−	+	−	−	+	−	459.18; 283.15; 265.14; 247.13; 219.14; 201.13	
Total metabolites						25	27	15	30	27	22

Note: “**+**” means detected; “**−**” means not detected. P represents prototype, DHA.

#### Structural Elucidation of DHA Metabolites

**FIGURE 5:**
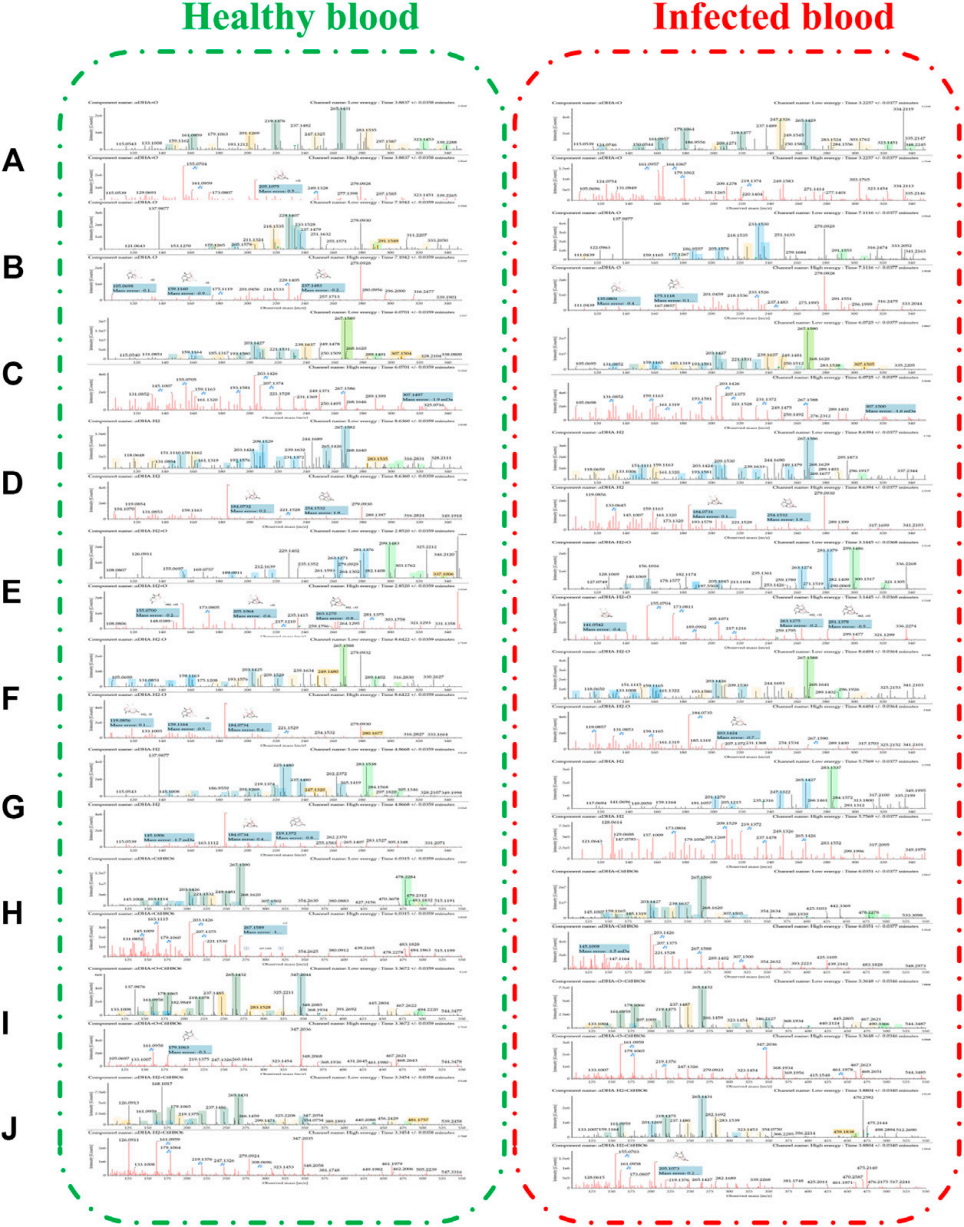
The spectra of metabolites in healthy blood samples (left) and infected blood samples (right): **(A)** DHA + O (M1–M7); **(B)** DHA-O (M8); **(C)** DHA-O + O (M9, M10); **(D)** DHA-H_2_ (M11); **(E)** DHA-H_2_+O (M12–M18); **(F)** DHA-H_2_-O (M19); **(G)** DHA-H_2_-O + O (M20–M28); **(H)** DHA + C_6_H_8_O_6_ (M29); **(I)** DHA + O + C_6_H_8_O_6_ (M30, M31); **(J)** DHA-H_2_+C_6_H_8_O_6_ (M32–M34).

All metabolites were detected within 16 min through a chromatographic method. Metabolites M1–M7 (DHA + O), eluted from 3.22 to 5.79 min, were proposed to be the result of hydroxylation of DHA. The quasimolecular ions were *m/z* 323 [M + Na]^+^ and *m/z* 339 [M + K]^+^. They showed a similar MS fragmentation pattern to that of DHA, except for a 16 Da mass shift (an oxygen atom). Fragment *m/z* 283 was generated by the loss of H_2_O from *m/z* 323 [M + Na]^+^. High-energy spectra showed that fragments *m/z* 265 and 247 were generated by the successive loss of H_2_O from *m/z* 283. Fragment *m/z* 219 was generated by the loss of HCOOH from *m/z* 265 (Figure 5).

Metabolite M8 (DHA-O), eluted at 7.09 min, was identified as 1 deoxy DHA. It was a known metabolite of DHA; the quasimolecular ions were *m/z* 291 [M + Na]^+^ and *m/z* 307 [M + K]^+^. This metabolite showed a similar MS fragmentation pattern to that of DHA. Fragments *m/z* 251 and 233 were generated by the successive loss of H_2_O from *m/z* 291. Fragment *m/z* 205 was generated by the loss of HCOOH from *m/z* 251 (Figure 5).

Metabolites M9-M10 (DHA-O + O), eluted from 4.17 to 6.04 min, were proposed to be the result of hydroxy-deoxygenation of DHA. Their quasimolecular ions were *m/z* 307 [M + Na]^+^ and *m/z* 323 [M + K]^+^. These were isomers of DHA and exhibited the same fragments as DHA, such as *m/z* 267, 249, 231, 221, and 203 (Figure 5).

Metabolite M11 (DHA-H_2_), eluted at 8.63 min, was identified as ART. It was a known metabolite of DHA; the quasimolecular ions were *m/z* 283 [M + H]^+^, *m/z* 305 [M + Na]^+^, and *m/z* 321 [M + K]^+^. Fragments *m/z* 265 and 247 were consistent with the reference substance (Figure 5).

Metabolites M12-M18 (DHA-H_2_+O), eluted from 2.48 to 5.10 min, were proposed to be the result of OH-dehydrogenation of DHA. Their quasimolecular ions were *m/z* 337 [M + K]^+^, *m/z* 321 [M + Na]^+^, and *m/z* 299 [M + H]^+^. Fragments *m/z* 281 and 263 were generated by the successive loss of H_2_O from *m/z* 299. Fragment *m/z* 235 was generated from *m/z* 281 following the loss of HCOOH (Figure 5).

Metabolite M19 (DHA-H_2_-O), eluted at 8.64 min, was identified as 1 deoxy ART. The quasimolecular ions were *m/z* 305 [M + K]^+^, *m/z* 289 [M + Na]^+^, and *m/z* 267 [M + H]^+^. Fragments m/z 249 and 231 were generated by the successive loss of H_2_O from *m/z* 267. Fragment *m/z* 221 was generated from *m/z* 267 following the loss of HCOOH (Figure 5).

Metabolites M20-–M28 (DHA-H_2_-O + O), eluted from 3.21 to 5.75 min, were proposed to be the result of OH-dehydration of DHA. Their quasimolecular ions were *m/z* 305 [M + Na]^+^ and 283 [M + H]^+^. They were isomers of ART, and their MS fragmentation pattern was consistent with that of ART. Fragments *m/z* 265, 247, and 229 were generated by the successive loss of H_2_O from *m/z* 283. In addition, fragment *m/z* 237 was generated by loss of HCOOH from *m/z* 283. Fragments *m/z* 219 and 201 were generated by the successive loss of H_2_O from *m/z* 237 (Figure 5).

Metabolite M29 (DHA + C_6_H_8_O_6_), eluted at 6.03 min, was predicted to be the result of glucuronidation of DHA. The quasimolecular ion was *m/z* 478 [M + NH_4_]^+^. Except for a 176 Da mass shift (a glucuronic acid molecular), fragments *m/z* 267, 249, 231, 221, and 203 were consistent with those of DHA (Figure 5).

In addition, metabolites M30-M31 (DHA + O + C_6_H_8_O_6_), eluted from 3.21 to 3.37 min, were predicted to be the result of glucuronidation of hydroxy-DHA. The quasimolecular ions were *m/z* 499 [M + Na]+ and *m/z* 494 [M + NH_4_]^+^. Except for a 176 Da mass shift (a glucuronic acid molecular), fragments *m/z* 283, 265, and 247 were consistent with the DHA-hydroxylated products (M1–M7) (Figure 5).

Metabolites M32–M34 (DHA-H_2_+C_6_H_8_O_6_), eluted from 3.23 to 3.88 min, were predicted to be the result of glucuronidation of dehydro-DHA. The quasimolecular ions were *m/z* 459 [M + H]^+^ and *m/z* 481 [M + Na]^+^. Except for a 176 Da mass shift (a glucuronic acid molecular), fragments *m/z* 265, 247, and 237 were consistent with ART (M11) and OH-dehydration of DHA (M20–M28) (Figure 5).

#### Comparison of DHA Metabolites in Infected and Healthy Mice

##### Blood Samples

The DHA metabolites in mouse blood samples were identified using the UNIFI platform. Except for DHA, a total of 25 (30) putative metabolites were identified in infected blood (healthy blood) [i-blood (h-blood)]. These comprised phase I metabolites: 7 (7) DHA + O, 1 (1) 1 deoxy DHA, 2 (2) DHA-O + O, 1 (1) ART, 4 (5) DHA-H_2_+O, 1 (1) 1 deoxy ART, and 7 (8) DHA-H_2_-O + O; and phase II metabolites: 1 (1) DHA + C_6_H_8_O_6_, 0 (2) DHA + O + C_6_H_8_O_6_, and 1 (2) DHA-H_2_+C_6_H_8_O_6_ (Table 2).

**TABLE 2:** Identified metabolites of DHA in blood samples of infected and healthy mice.

Metabolite type	Infected mouse			Healthy mouse		
	Blood	Plasma	RBC	Blood	Plasma	RBC
DHA	1	1	1	1	1	1
DHA + O	7	7	4	7	6	7
DHA-O	1	1	0	1	1	1
DHA-O+O	2	2	2	2	2	2
DHA-H_2_	1	0	0	1	0	0
DHA-H_2_+O	4	4	2	5	5	2
DHA-H_2_-O	1	1	1	1	1	1
DHA-H_2_-O+O	7	8	4	8	8	7
DHA + C_6_H_8_O_6_	1	1	1	1	1	1
DHA + O + C_6_H_8_O_6_	0	2	0	2	2	0
DHA-H_2_+C_6_H_8_O_6_	1	1	1	2	1	1
Total metabolites	25	27	15	30	27	22

The results were compared with related reference substances, which were isolated and identified from microbial transformation products and degradation products of ART derivatives in our laboratory. According to the retention time and MS fragmentation, several metabolites were identified in i-blood and h-blood, such as 8-OH DHA, 1 deoxy DHA, 3*α*-OH deoxy DHA, ART, 1 deoxy ART, 4*α*-OH deoxy ART, and 6*β*-OH deoxy ART (Table 3).

**TABLE 3:** Well-defined metabolites of DHA in blood samples of infected and healthy mice.

Identified metabolite	Infected mouse			Healthy mouse		
	Blood	Plasma	RBC	Blood	Plasma	RBC
DHA	+	+	+	+	+	+
8-OH DHA	+	+	−	+	+	+
1 deoxy DHA	+	+	−	+	+	+
3*α*-OH deoxy DHA	+	+	+	+	+	+
ART	+	−	−	+	−	−
1 deoxy ART	+	+	+	+	+	+
4*α*-OH deoxy ART	+	+	+	+	+	+
6*β*-OH deoxy ART	+	+	+	+	+	+

Note: “+” means detected; “-” means not detected.

##### Plasma Samples

The DHA metabolites in plasma samples were identified using the UNIFI platform. Except for DHA, a total of 27 (27) putative metabolites were identified in infected plasma (healthy plasma). These comprised phase I metabolites: 7 (6) DHA + O, 1 (1) 1 deoxy DHA, 2 (2) DHA-O + O, 4 (5) DHA-H_2_+O, 1 (1) 1 deoxy ART, and 8 (8) DHA-H_2_-O + O; and phase II metabolites: 1 (1) DHA + C_6_H_8_O_6_, 2 (2) DHA + O + C_6_H_8_O_6_, and 1 (1) DHA-H_2_+C_6_H_8_O_6_ (Table 2).

According to the retention time and MS fragmentation of related reference substances in our laboratory, several metabolites were identified in infected and healthy plasma, such as 8-OH DHA, 1 deoxy DHA, 3*α*-OH deoxy DHA, 1 deoxy ART, 4*α*-OH deoxy ART, and 6*β*-OH deoxy ART (Table 3).

##### RBC Samples

The DHA metabolites in RBC samples were identified using the UNIFI platform. Except for DHA, a total of 15 (22) putative metabolites were identified in i-RBC (h-RBC). These comprised phase I metabolites: 4 (7) DHA + O, 0 (1) 1 deoxy DHA, 2 (2) DHA-O + O, 2 (2) DHA-H_2_+O, 1 (1) 1 deoxy ART, and 4 (7) DHA-H_2_-O + O; phase II metabolites: 1 (1) DHA + C_6_H_8_O_6_ and 1 (1) DHA-H_2_+C_6_H_8_O_6_ (Table 2).

According to the retention time and MS fragmentation of related reference substances in our laboratory, several metabolites were identified in both i-RBC and h-RBC, such as 3*α*-OH deoxy DHA, 1 deoxy ART, 4*α*-OH deoxy ART, and 6*β*-OH deoxy ART. Moreover, 8-OH DHA and 1 deoxy DHA were identified inside h-RBC (Table 3).

## Discussion

**FIGURE 6:**
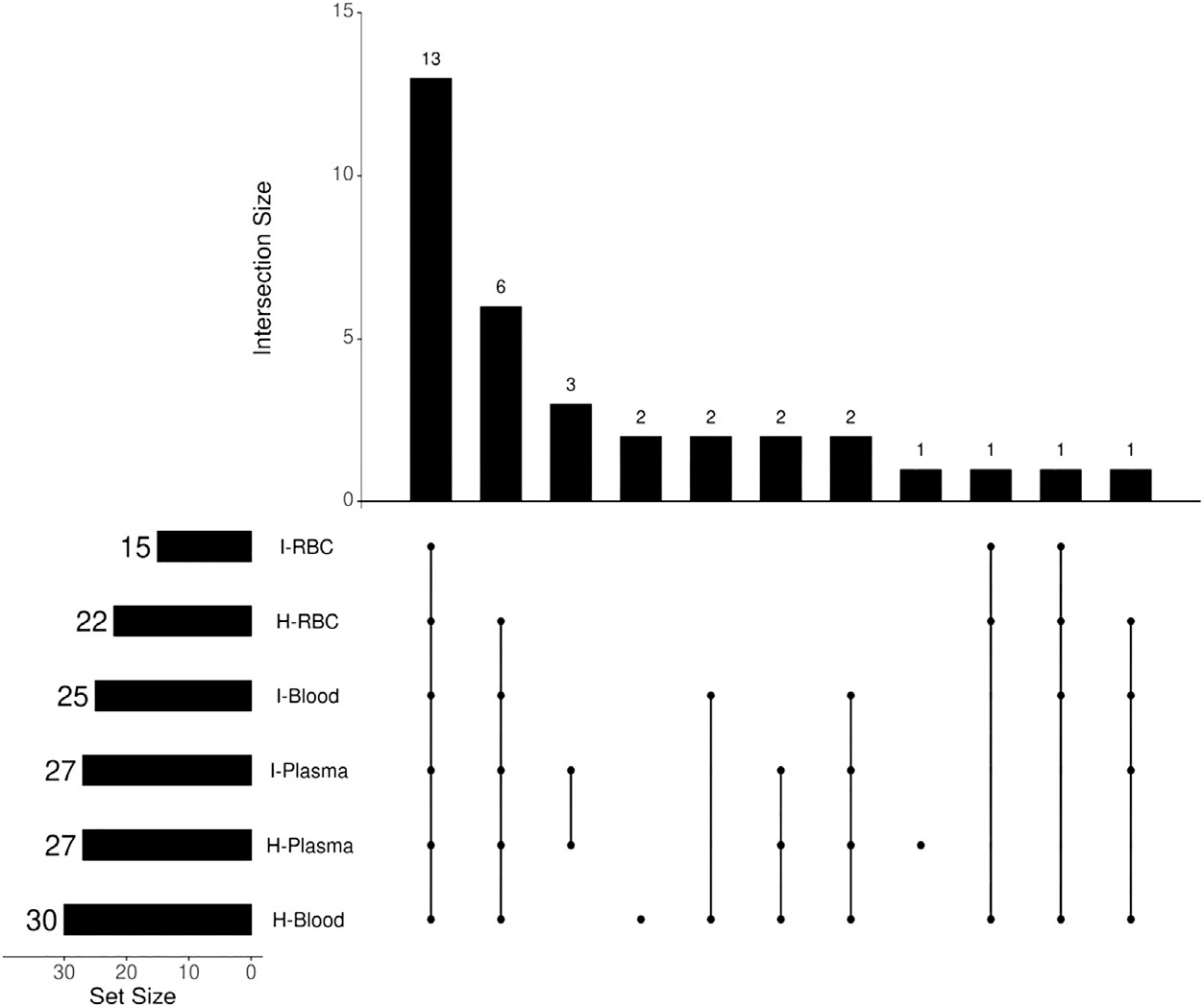
Diagram of the number of intersections of DHA metabolites in mouse blood samples.

The antimalarial mechanism of ART-based drugs is a global concern and remains controversial. The metabolism of drugs *in vivo* is closely related to the antimalarial mechanism. Most previous studies on the metabolism of ART drugs were performed on healthy animals, rather than *Plasmodium*-infected model. Thus, a C57 mouse model infected by *Plasmodium berghei* ANKA strain was established, and a comparison of DHA metabolism between infected and healthy mice was conducted in this study. The results showed that a total of 34 DHA metabolites were identified *in vivo*. DHA metabolites among different biosamples are similar (Figure 6). There were also differences noted between infected and healthy mice. The number of DHA metabolites in i-blood was significantly less than that recorded in h-blood, especially for phase II metabolites. The relative content (peak area) of the main metabolites between infected samples and healthy samples was significantly different, indicating that *Plasmodium* had damaged the liver metabolic function of mice.

It is known that the *Plasmodium* species parasitize inside RBC, but the detection of DHA metabolites inside RBC is a great challenge. A previous study (Tang et al., 2019) has shown that i-RBC is rich in heme, hemin, and free iron ions (Fe^2+^ and Fe^3+^), which are digested from hemoglobin by *Plasmodium*. Following entry into i-RBC, the drug is easily reduced by heme, free iron ions, and other components (Zhang and Gerhard, 2008). It is difficult to track the metabolites produced by nonenzymatic biotransformation; hence, the addition of chelating agents to RBC is necessary. In this study, chelating agent and oxidizing agent were employed for the chelation of Fe^2+^ and Fe^3+^ to protect the structures of DHA metabolite. This is the first attempt to identify DHA metabolites inside RBC and blood samples. The results showed that the established method successfully identified 15 (22) putative metabolites in i-RBC (h-RBC). Previous studies have demonstrated that radioisotope-labeled 3H-DHA was accumulated from the culture medium into RBC, especially in i-RBC. However, the number of DHA metabolites and the relative content (peak area) of main metabolites inside i-RBC were significantly lower than those recorded in h-RBC, especially the products of hydroxylation of DHA (DHA + O) and OH-dehydration of DHA (DHA-H_2_-O + O). Combined with our results, it was hypothesized that DHA and bioactive metabolites immediately bind to the target, result in nonenzymatic biotransformation, or disrupt the metabolic balance of *Plasmodium*, after entering i-RBC and exerting their effect. Therefore, further qualitative and quantitative analyses of DHA and its metabolites inside RBC are warranted.

**FIGURE 7:**
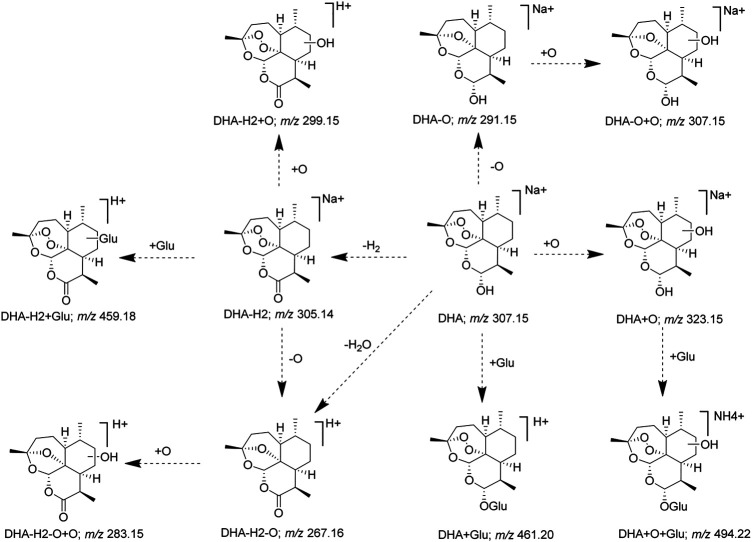
Proposed metabolic pathways of DHA.

Furthermore, according to the identified DHA metabolites *in vivo*, the hydroxylation, OH-dehydration, and glucuronidation reactions were found to be important in the metabolic pathway, and the metabolic pathway of DHA was constructed (Figure 7). Some DHA metabolites (OH DHA, ART, glucuronidation of DHA, etc.) were considered to possess antimalarial activity. The active metabolites of DHA and the prototype drug play an antimalarial effect synergistically.

Finally, combined with the existing related reference substances in our laboratory, seven metabolite structures were identified based on the chromatographic retention time and MS fragmentation pattern (Table 3). Among them, 8-OH DHA, 4*α*-OH deoxy ART, and 6*β*-OH deoxy ART were newly identified DHA metabolites; ART and 8-OH DHA possess antimalarial activity.

## Conclusion

In summary, a C57 mouse model infected by *Plasmodium berghei* ANKA strain was established, and a comparison of DHA metabolites in infected and healthy mice was also performed. The UPLC-Q-TOF-MS^E^ method and UNIFI platform were successfully employed to identify the metabolites of DHA *in vivo*. Meanwhile, a chelating agent and an oxidizing agent were employed to protect the structures of DHA metabolites, and DHA metabolites inside RBC were identified for the first time. The hydroxylation, OH-dehydration, and glucuronidation reactions were found to be important in the metabolic pathway, and *Plasmodium* damaged the liver metabolic function of mice. Subsequently, the metabolic pathway of DHA *in vivo* was constructed. It was hypothesized that DHA and bioactive metabolites immediately bind to the target or disrupt the metabolic balance of *Plasmodium*, after entering i-RBC and exerting their effect. Furthermore, combined with reference substances in our laboratory, seven metabolite structures were identified. Notably, 8-OH DHA, 4*α*-OH deoxy ART, and 6*β*-OH deoxy ART were newly identified DHA metabolites. Some DHA metabolites (OH DHA, ART, glucuronidation of DHA, etc.) possess antimalarial activity. Collectively, the results laid a solid foundation for further pharmacological research and offered new insight into the antimalarial mechanism of ART drugs.

## Data Availability Statement

The raw data supporting the conclusions of this article will be made available by the authors, without undue reservation.

## Ethics Statement

The animal study was reviewed and approved by the Ethics Committee of the China Academy of Chinese Medical Sciences (ethics approval number: 2019080).

## Author Contributions

LY and DZ conceived and designed the experiments. YZ and PS performed the experiment. YM and YB prepared the reference substances. XQC, XYC, and XJ analyzed the data. All the authors gave the final approval of the version to be submitted.

## Funding

This work was supported by the Emergency Management Program of National Natural Science Foundation of China (Grant No. 81841001) and major science and technology project for “Signiﬁcant New Drugs Creation” (Grant No. 2017ZX09101002-002).

## Conflict of Interest

The authors declare that the research was conducted in the absence of any commercial or financial relationships that could be construed as a potential conflict of interest.
